# A Device-Independent Evaluation of Carbonyl Emissions from Heated Electronic Cigarette Solvents

**DOI:** 10.1371/journal.pone.0169811

**Published:** 2017-01-11

**Authors:** Ping Wang, Wenhao Chen, Jiawen Liao, Toshiki Matsuo, Kazuhide Ito, Jeff Fowles, Dennis Shusterman, Mark Mendell, Kazukiyo Kumagai

**Affiliations:** 1 Indoor Air Quality Program, Environmental Health Laboratory, California Department of Public Health, Richmond, California, United States of America; 2 Division of Environmental Health Sciences, School of Public Health, University of California, Berkeley, California, United States of America; 3 Interdisciplinary Graduate School of Engineering Sciences, Kyushu University, Kasuga, Fukuoka, Japan; 4 Exposure Assessment Section, Environmental Health Investigation Branch, California Department of Public Health, Richmond, California, United States of America; 5 Division of Occupational and Environmental Medicine, School of Medicine, University of California, San Francisco, California, United States of America; Louisiana State University, UNITED STATES

## Abstract

**Objectives:**

To investigate how the two main electronic (e-) cigarette solvents—propylene glycol (PG) and glycerol (GL)—modulate the formation of toxic volatile carbonyl compounds under precisely controlled temperatures in the absence of nicotine and flavor additives.

**Methods:**

PG, GL, PG:GL = 1:1 (wt/wt) mixture, and two commercial e-cigarette liquids were vaporized in a stainless steel, tubular reactor in flowing air ranging up to 318°C to simulate e-cigarette vaping. Aerosols were collected and analyzed to quantify the amount of volatile carbonyls produced with each of the five e-liquids.

**Results:**

Significant amounts of formaldehyde and acetaldehyde were detected at reactor temperatures ≥215°C for both PG and GL. Acrolein was observed only in e-liquids containing GL when reactor temperatures exceeded 270°C. At 318°C, 2.03±0.80 μg of formaldehyde, 2.35±0.87 μg of acetaldehyde, and a trace amount of acetone were generated per milligram of PG; at the same temperature, 21.1±3.80 μg of formaldehyde, 2.40±0.99 μg of acetaldehyde, and 0.80±0.50 μg of acrolein were detected per milligram of GL.

**Conclusions:**

We developed a device-independent test method to investigate carbonyl emissions from different e-cigarette liquids under precisely controlled temperatures. PG and GL were identified to be the main sources of toxic carbonyl compounds from e-cigarette use. GL produced much more formaldehyde than PG. Besides formaldehyde and acetaldehyde, measurable amounts of acrolein were also detected at ≥270°C but only when GL was present in the e-liquid. At 215°C, the estimated daily exposure to formaldehyde from e-cigarettes, exceeded United States Environmental Protection Agency (USEPA) and California Office of Environmental Health Hazard Assessment (OEHHA) acceptable limits, which emphasized the need to further examine the potential cancer and non-cancer health risks associated with e-cigarette use.

## Introduction

E-cigarettes, as battery-powered nicotine delivery devices with many different configurations, have increased in popularity in recent years, especially among youth. According to the California State Health Officer’s 2015 report [1], e-cigarette use among teens surpassed the use of traditional cigarettes for the first time in 2014, and the prevalence of such use tripled in one year from 2.3% to 7.6% in 2013 for young adults (18–29 years old, including 20% who have never tried traditional cigarettes). Nationally, 8.1% of adults have tried this new device, with 1.4% being current users in 2012 [2]. In order to fuel the rapidly growing market for e-cigarettes, manufacturers advertise these new devices as healthier and safer alternatives to traditional tobacco smoking or as a tool for smoking cessation. However, in addition to the questionable premise that e-cigarettes assist in smoking cessation, increasing scientific evidence indicates that e-cigarettes produce more than “harmless water vapor” during “vaping”. Immediate adverse physiological effects after short-term use of e-cigarettes have been reported. For example, Vardavas et al. [3] observed pulmonary effects similar to those seen with tobacco smoking after using e-cigarettes for five minutes. Flouris et al. [4] found that e-cigarettes caused measurable (but smaller) changes in lung function compared to combusted tobacco, while short-time e-cigarettes use produced a similar nicotinergic impact. The potential human health risks posed by the long-term use of e-cigarettes directly on vapers and indirectly on bystanders, from second-hand smoking as well as third-hand exposures, remain largely unknown [5]. Therefore, as use of e-cigarettes increases rapidly, more studies on e-cigarettes are urgently needed to ensure that consumers’ (and bystanders’) health is safeguarded.

Although e-cigarettes have a very wide variation in terms of device design and component functionality, they typically share basic common features, which include an aerosol generator (heating element), a flow sensor, a battery, and a storage tank for a nicotine-containing solution (usually called e-liquid or e-juice) [6]. In order to generate aerosols, the vaper inhales at the mouthpiece of e-cigarettes to allow e-liquid, generally containing PG or GL (or a mixture of both) as nicotine solvent, along with or without nicotine, water, and flavorants added for various tastes, to pass through the heating element and to be vaporized. Of increasing concern are the thermal breakdown products from e-liquids at high temperature, due either to the device design or to unintentional overheating. For example, in some “voltage adjustable” e-cigarette devices, users can raise the battery output voltage to generate more aerosols and to increase nicotine delivery through increased internal temperature. In fact, the achievable temperature inside e-devices ranges up to 350°C [7, 8], sufficiently high to decompose PG or GL. Diaz et al. [9] studied the homogeneous oxidation reaction of PG in a 10% O_2_/He mixture at different temperatures and concluded that PG can be easily oxidized at as low as 127–227°C to form formaldehyde, acetaldehyde, and carbon dioxide via oxidative C-C cleavage and to form acetone via a dehydration route. The dehydration of GL to yield acrolein is another well-known reaction in organic chemistry. Acrolein can undergo further degradation to form formaldehyde and acetaldehyde or other small molecules depending on circumstances [10]. For instance, carbon monoxide, acetaldehyde, and acrolein were detected as the initial products by pyrolysis of GL through a laminar flow reactor [11].

Among the reported thermal breakdown products from PG and GL, low molecular weight carbonyls such as formaldehyde, acetaldehyde, and acrolein have drawn much attention due to their known toxicity. Formaldehyde is a Group 1 human carcinogen as classified by the International Agency for Research on Cancer (IARC), and acetaldehyde is regarded as a Group 2B possible human carcinogen [12]. Acrolein is toxic, is a strong irritant for the skin, eyes, and nasal passages, and is on the original list of hazardous air pollutants from the United States Environmental Protection Agency (U.S. EPA) [13]. To date, a number of papers [7] have reported the presence of low molecular weight carbonyl compounds and two additional potentially harmful compounds, propylene oxide and glycidol, by Sleiman et al. [14] from e-cigarette aerosols. Very recently, Geiss et al. [15] studied the correlation of volatile carbonyl yields with the heating coil temperature using a third generation of e-cigarette device, an increasingly popular model with adjustable output power. However, because of limited understanding of the role played by critical variables, e.g., device type, e-liquid composition, and user’s puffing behavior, in terms of emitted toxic compounds, the measured carbonyl concentrations from e-cigarettes have a very large variability among different studies. Some factors that could affect measured amount of carbonyls are summarized in Table 1. Furthermore, it is quite challenging to accurately determine the operating coil temperature during actual e-cigarette vaping. A temperature difference as high as 100°C was observed by Geiss et al. [15] between the first and the last puffs in five consecutive puffs. Given the differences in commercial e-liquids and e-cigarette design and use, a standardized method is necessary to evaluate carbonyl emissions from e-cigarette vaping at specific temperatures.

**Table 1:** Variables that could affect measured amount of carbonyls.

e- device design	power output (voltage/wattage), heating coil element, coil resistance, single or double coil, wick material, air hole, heating coil position
e-liquid composition	propylene glycol, glycerol, flavor, nicotine, impurities, other possible additives, and their concentrations
vaper's topography	puff duration, puff interval, puff volume, puff number
methodology used by authors	smoking machine, human subject, or other home-made setup, carbonyl collection and analytical method, level of liquid inside e-device, battery charging level

In this study, we employed a convenient tube reactor to mimic e-cigarette vaping under precisely controlled temperatures. The aim was to investigate how the two main e-cigarette solvents—propylene glycol and glycerol—modulate thermal breakdown to toxic carbonyls in emitted aerosols, without using any specific e-cigarette device type and in the absence of other additives, e.g., nicotine and flavoring. We hypothesize that any differences between thermal breakdown products from PG and GL would contribute to our fundamental understanding of the source of toxic carbonyls from e-cigarette use. Knowing the link between amount of generated carbonyls from different e-cigarette solvents and precisely measured temperature will provide important insights of toxicological risk exposure assessment from e-cigarette use.

## Methods

### E-liquids

Five e-liquids were tested at 50, 100, 150, 200, 250 and 300°C (reactor’s skin temperature): PG (>99.5%, Sigma-Aldrich, USA) without further purification before use; GL (>99.5%, Sigma-Aldrich, USA) without further purification before use; PG:GL = 1:1 (wt/wt) mixture; and two commercial e-liquids (#1, “tobacco” flavor, purchased from a supermarket and #2, “MIN TEE” flavor, purchased from a local “vaping bar” store in California). The main ingredients in the two commercial e-liquids were both labelled “propylene glycol and glycerol” without information on concentrations or proportions. The label on the 15-ml bottle of e-liquid #1 listed the nicotine content as 12 mg; while the label on e-liquid #2 stated that it “may have nicotine inside” with no further information. A refillable cartridge and device were also purchased with e-liquid #2 to measure chemical emission rate. In order to estimate human exposures during actual e-cigarette vaping at different temperatures, the amount of commercial e-liquid #2 consumed by ten 50-ml puffs was measured using a gas-tight syringe. The puff duration was 3-s. For each combination of e-liquid and temperature, at least two parallel experiments were conducted and the results were averaged.

### Vaporization of e-liquid

**Fig 1:**
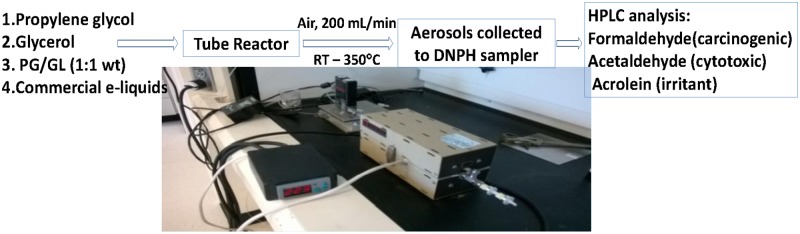
Experiment setup and schematic diagram for carbonyl measurement.

The experiment setup and schematic diagram were shown in Fig 1. To vaporize e-liquid, a precisely weighed 5–10 mg of e-liquid was loaded at the center of a 0.3-g piece of glass wool, which was then carefully transferred to the middle of a stainless steel, tubular reactor (25 cm long and 1 cm in inner diameter) making sure the test liquid did not contact inner surface of the reactor. The reactor was housed in a horizontal, split-sided furnace. A temperature controller (Model SDC120JF-A, Brislheat Corp., USA) was used to regulate skin temperature of the reactor. The temperature of glass wool inside the reactor was denoted as reactor temperature. It was measured with a thermocouple (EL-USB-TC-LCD, DATAQ Instruments Inc., USA). One end of the tube reactor was connected to compressed air with a constant flow rate of 200 mL/min regulated by a mass flow controller (Smart-Track Digital Mass Flow Meter, Sierra Instruments, Inc., USA). At this flow rate, the transition time of e-liquid with air in the reactor was 2.9 s in order to mimic a ~3-s puff duration. Aerosols were collected onto commercial cartridge samplers that contained silica coated with 2, 4-dinitrophenylhydrazine (DNPH) (Sep-Pak XpoSure Aldehyde Sampler, WAT047025, Waters, Inc., USA). The temperature of air leaving the reactor was between 25–76°C, depending on the reactor temperature. Hence there was no concern that a high temperature of leaving air from the reactor would damage the DNPH cartridges. Two DNPH cartridges were connected in series to ensure no cartridge breakthrough.

Before each experiment, the reactor was first thoroughly cleaned with deionized water and heated at 350°C with flowing air for 3 hours to remove organic residuals. Then the background level of carbonyls from a 0.3-g clean piece of glass wool was measured by inserting it into the middle of the reactor and collecting an effluent gas sample for one hour at the desired test temperature. Finally, a new piece of glass wool loaded with e-liquid was inserted into the reactor and heated at desired temperature to generate aerosols. Sampling time of DNPH cartridge was one hour. The amount of carbonyls measured after one hour was negligible. The corresponding background level of carbonyls was subtracted from all results.

### Analysis of carbonyl compounds

The DNPH cartridges were extracted with 10 mL of acetonitrile (HPLC grade, Fisher, USA) in a 10-ml volumetric flask, and 1.0 mL of the extract was transferred to an HPLC sample vial. HPLC analysis was performed on an Agilent 1260 HPLC with a UV-DAD detector measured at 360 nm (Agilent Technology, Inc., USA) under isocratic mode. A 200-mm × 3.2-mm C18 column (Restek Allure, USA) was used at 40°C. The mobile phase was acetonitrile/water (65v/35v) with a flow rate at 0.75 mL/min. Carbonyl compounds were identified and quantified according to commercial standards (Sigma-Aldrich, USA). The method detection limits for formaldehyde, acetaldehyde, acrolein, and acetone were 0.007, 0.010, 0.012, and 0.012 μg/mL, respectively.

### Statistical analyses

To compare generation of formaldehyde and acetaldehyde from the pure PG and GL carriers, the comparison of primary interest in this study, we used an unpaired *t*-test with a two-sided significance level of 0.05 at two specific temperatures: 270 and 318°C.

## Results and Discussion

### Formation of carbonyl compounds from propylene glycol (PG)

**Fig 2:**
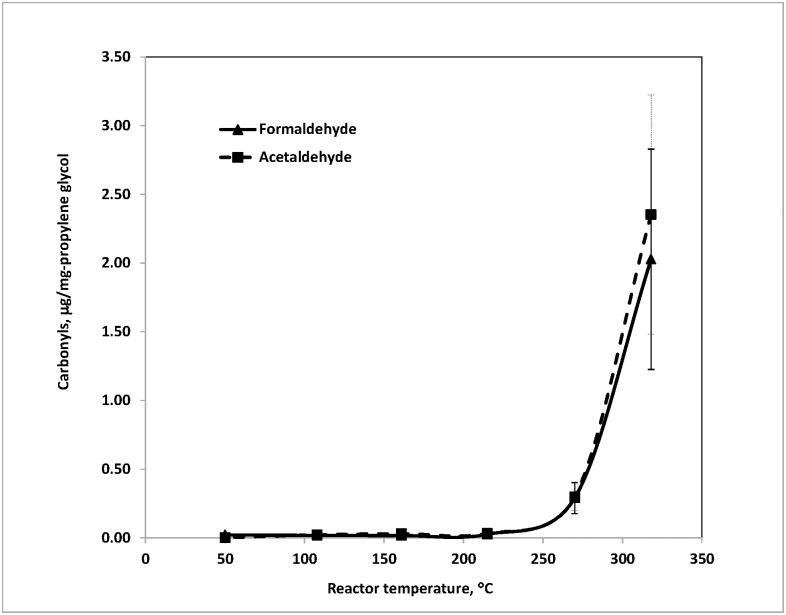
Carbonyl compounds generated from propylene glycol at various temperatures in flowing air at 200 mL/min. Amount of propylene glycol: ~5 mg. Each temperature repeated 2–8 times.

Fig 2 shows the amounts of formaldehyde and acetaldehyde, expressed in micrograms generated from per milligram of loaded PG, at various heating temperatures. Besides these two aldehydes, a trace amount of acetone was also observed but is not plotted in the figure. The results are consistent with the findings of PG thermal oxidation reported by Diaz et al. [9] and Bekki et al. [16]. When the reactor temperature reached 215°C, formaldehyde and acetaldehyde began to increase noticeably. The quantity of formaldehyde was 0.03±0.03 μg/mg-PG at 215°C, climbing steeply to 0.29±0.11 μg/mg-PG at 270°C, and reaching as high as 2.03±0.80 μg/mg-PG at 318°C.

Emission of acetaldehyde occurred at the same temperatures as emission of formaldehyde, as expected from their concurrent formation via a C-C bond cleavage of a C3 molecule. The quantity of acetaldehyde was 0.03±0.00 μg/mg-PG at 215°C, 0.30±0.11 μg/mg-PG at 270°C, and 2.35±0.87 μg/mg-PG at 318°C. Formation of a trace amount of acetone was likely due to the dehydration of PG. Moreover, as the amount of loaded PG increased, the amount of formed aldehydes increased almost linearly.

These results suggest that the total amount of carbonyls emitted from e-cigarettes is not only closely associated with the vaping temperature but also with the e-liquid mass consumed by vapers. Kosmider et al. [17] reported that increasing voltage from 3.2 to 4.8 V in a “voltage adjustable” e-cigarette resulted in formaldehyde, acetaldehyde, and acetone levels increasing by 4 to >200 times. In their study, formaldehyde and acetaldehyde yields reached 17.6±19.7 μg and 4.2±3.2 μg per 15 puffs (70 mL/puff), respectively. Using a headspace GC-MS to incubate e-liquid at different temperatures, Hutzler et al. [7] found that the amount of formaldehyde and acetaldehyde was up to 10- to 20-fold higher at 150°C when compared to ambient temperature for an e-liquid containing PG as a main component. The authors also noticed that the formation of a significant level of carbonyls appeared only in the second half of their smoking protocol, i.e., not before puff 60. They attributed this observation to overheating as the e-liquid level decreased with consumption. 20 to 50 micrograms of formaldehyde were measured per 10 puffs during their final fractions. This level of exposure was roughly comparable to smoking one conventional cigarette. For this reason, prevention of high temperature and overheating during vaping to minimize the formation of toxic chemicals is a design feature that must be considered to safeguard public health.

### Formation of carbonyl compounds from glycerol (GL)

**Fig 3:**
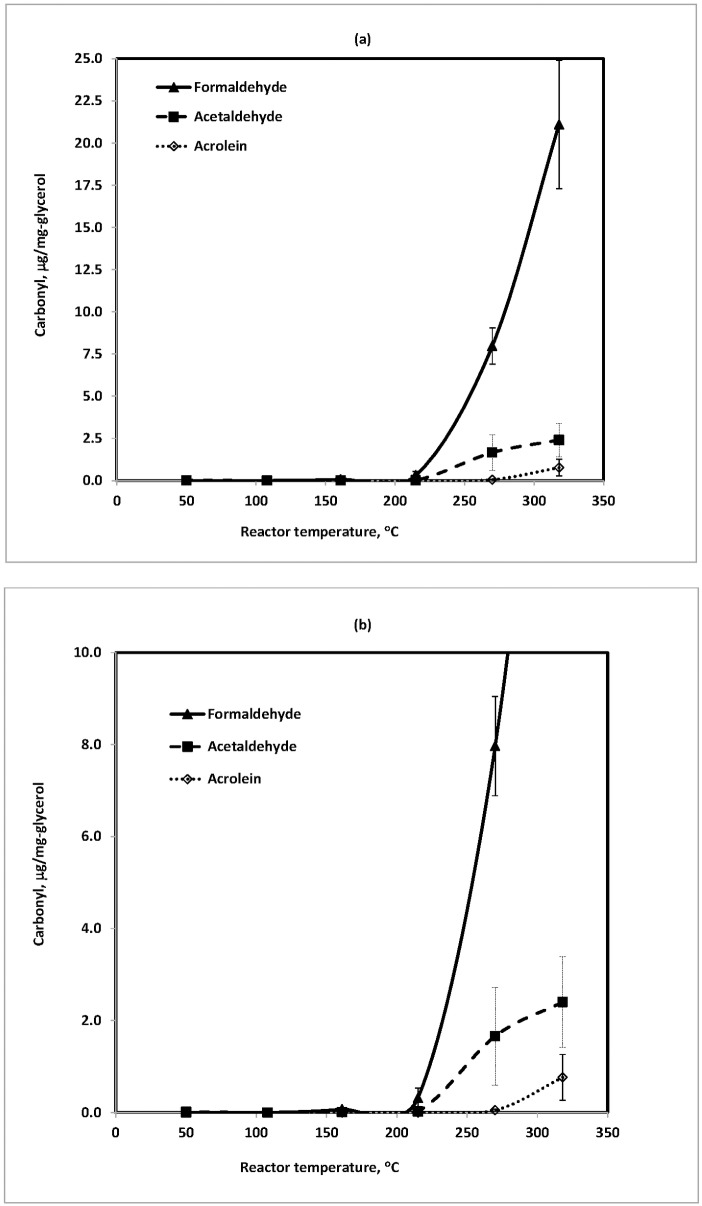
Carbonyl compounds generated from glycerol (~10 mg) at various temperatures in flowing air at 200 mL/min: (a) full scale; and (b) enlarged scale. Amount of glycerol: ~10 mg. Each temperature repeated 2–5 times.

Glycerol (GL), also called glycerin or glycerine, was tested under the same conditions as PG. As displayed in Fig 3a, a similar temperature effect on the formation of formaldehyde and acetaldehyde was observed. However, with GL, unlike with PG, acrolein was also observed at detectable levels at reactor temperatures ≥270°C. It has been reported that acrolein, formaldehyde, and acetaldehyde were major products when GL was pyrolyzed in steam [11, 18] and supercritical water [19, 20]. Using quantum mechanical calculations, Nimlos et al. [10] proposed mechanisms for the formation of these compounds, and confirmed their findings using a triple-quadrupole mass spectrometer.

In this study, the amounts of formaldehyde, acetaldehyde, and acrolein from GL were 21.10±3.80, 2.40±0.99, and 0.80±0.50 μg/mg-GL at 318°C, respectively. It is noteworthy that the level of formaldehyde was about ten times higher from GL than from PG at the same temperature. Furthermore, the evolution of formaldehyde and acetaldehyde began at relatively lower temperatures (108 and 215°C, respectively), while the development of acrolein began at a relatively higher temperature (~270°C) (Fig 3b). With PG, but not GL, formaldehyde and acetaldehyde were initially formed almost simultaneously at ~200°C. This suggests that the thermal oxidation of PG must have different kinetics than GL, likely because of GL’s unique three adjacent hydroxyl groups in the C3 molecule. Kosmider et al. [17] observed a higher level of carbonyls from a PG-based solution and suggested that PG in e-cigarettes is more susceptible to thermal decomposition than GL. Sleiman et al. [14] and Geiss et al. [15] reported similar results with the present study. A possible explanation for the differences in reported carbonyl formation from GL is its potential interaction with reactor materials. Our study used a stainless steel reactor but no information of heating material was available in other studies. Considering e-device manufacturers use different metals as heating elements, future studies should examine the effect of different heating metals.

### Comparison of carbonyl compound generation from PG and GL

At 270°C, significantly more formaldehyde (over 27 times as much) was generated from GL than from PG (7.96 vs. 0.29 μg/mg-GL; t-value = -24.4, p-value<0.00001); significantly more acetaldehyde (over five times as much) was also generated from GL than from PG (1.66 vs. 0.30 μg/mg-GL; t-value = -4.5, p-value = 0.002). At 318°C, significantly more formaldehyde (over 10 times as much) was generated from GL than from PG (21.14 vs. 2.03 μg/mg-GL; t-value = -10.1, p-value = 0.0002); similar amounts of acetaldehyde were generated from GL and PG (2.37 vs, 2.35 μg/mg-GL; t-value = -0.025, p-value = 0.98). As we mentioned above with respect to the results discussed in Fig 3, this also indicated that thermal oxidation of GL must have different kinetics than PG, especially at higher temperature.

### Formation of carbonyl compounds from 1:1 weight ratio PG/GL mixture

**Fig 4:**
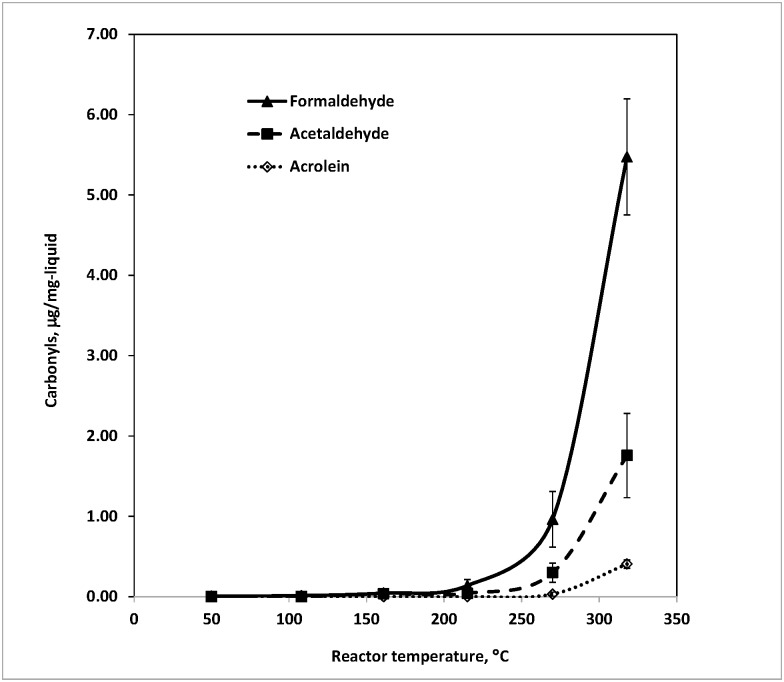
Carbonyl compounds generated from 1:1 weight ratio of propylene glycerol and Glycerol mixture (~5 mg) at various temperatures in flowing air at 200 mL/min. Each temperature repeated 2–5 times.

In order to achieve a balance of flavor intensity, throat “hit” (irritancy), and vapor density, the majority of e-liquids contain a solvent mixture of different proportions of PG and GL, rather than either alone. To determine if the thermal breakdown products of these mixtures may be different than those from single compounds, a solvent mixture with a one-to-one weight ratio of PG and GL was also tested at different temperatures. The overall trends of carbonyl generation with increasing reactor temperature were more similar to those of GL than those of PG, as shown in Fig 4.

In addition to formaldehyde and acetaldehyde, acrolein also became detectable at ~270°C, which agrees very well with the GL findings (Fig 3). This implies that the presence of acrolein in e-cigarette emissions mainly comes from GL decomposition, which can also explain the wide range of acrolein concentrations measured by different laboratories. If an e-liquid contained no GL, acrolein likely would not be produced even at high temperatures. On the other hand, the level of carbonyls generated from the PG/GL mixture is apparently not simply the sum of carbonyls generated from single PG and single GL. More complicated reactions, such as dehydration between PG and GL, might occur when the mixture is exposed to high temperatures.

### Formation of carbonyl compounds from commercial e-liquids

**Fig 5:**
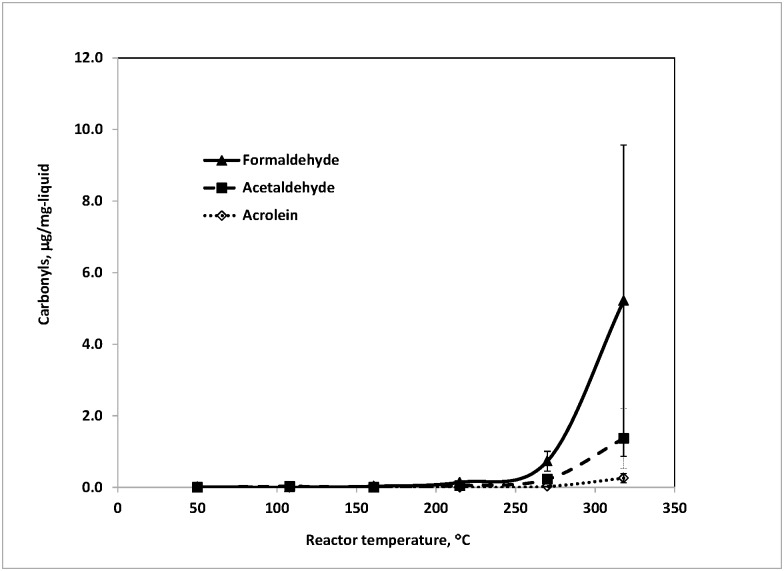
Carbonyls generated from commercial e-liquid #2 at various temperatures in flowing air at 200 mL/min. E-liquid amount: ~7 mg. Each temperature repeated 2–5 times.

Fig 5 illustrated the carbonyl results of commercial e-liquid #2 as an example. As expected, the emission levels of formaldehyde and acetaldehyde generally grew fast with increasing reactor temperatures. Acrolein became detectable at ~270°C, which coincided with our observation for GL alone as well as the PG/GL mixture. This result further confirmed that acrolein mainly resulted from the thermal decomposition of GL, although the formation of acrolein from flavorings and other additives in e-liquid cannot be excluded. No additional carbonyl compounds were detected even with the presence of additional flavors and nicotine in the two commercial e-liquids. PG and GL were likely to be the primary sources of emitted carbonyls from these two e-liquids. Confirming if this is true for e-liquids in general will require further research. Khlystov et al. [21] have observed that thermal decomposition of selected flavoring compounds dominated formation of several aldehydes during vaping in their study.

The levels of carbonyl compounds generated from all five tested e-liquids at 270 and 318°C are summarized in Table 2 for comparison. Pure GL generated much higher amounts of formaldehyde than the other e-liquids. In contrast, pure PG generated the lowest amount of formaldehyde, and more complicated reactions occurred with the PG/GL mixture, especially at 318°C, as reflected by the change in molar ratio of formaldehyde to acetaldehyde. Chen et al. [7] and Geiss et al. [15] have summarized the amounts of carbonyls from e-cigarettes measured by different studies, which have varied widely. As listed in Table 1, many factors could affect final carbonyl emissions. Existing studies on the impact of e-liquid composition on carbonyls formation have not been fully conclusive: some observed higher emissions from pure PG [17], some observed higher emissions from pure GL [14, 15], while others found no significant correlation between carbonyl emissions and e-liquid composition (i.e., PG/GL ratio, nicotine concentration, water concentration, and pH value) when testing various e-liquids and e-cigarette devices [22]. Unlike our current study, none of these studies were conducted under precisely controlled and known temperatures. Our current study, for the first time, clearly demonstrated that PG and GL could generate different amounts of carbonyls even under exactly the same temperature and flow conditions, proving that e-liquid composition, especially the solvent type, can be one of the critical factors that affect e-cigarette emissions. Vaping topography may also impact emissions and aerosol generations from e-cigarettes [23], although this is not the focus of our current study. Tallish S. et al. [24] have investigated the effects of user puff topography on e-cigarette nicotine yield. They found that longer puffs resulted in higher nicotine yields while puff velocity had no effect on nicotine yield. However, whether these conclusions can extend to carbonyl compound emissions needs further research.

**Table 2:** Carbonyls generated at 270 and 318°C from different e-liquids.

E-liquid	Carbonyls generated at 270°C, μg/mg-liquid			
	Formaldehyde	Acetaldehyde	Acrolein	Formaldehyde/Acetaldehyde (mole/mole)
PG	0.29 ± 0.11	0.30 ± 0.10	ND	1.4
GL	7.97 ± 1.08	1.70 ± 1.06	0.05 ± 0.02	6.9
PG/GL (1:1)	0.96 ± 0.35	0.30 ± 0.12	0.03 ± 0.02	4.7
E-liquid #1	0.97 ± 0.87	0.20 ± 0.15	0.05 ± 0.04	7.1
E-liquid #2	0.73 ± 0.28	0.22 ± 0.09	0.02 ± 0.00	4.9
Carbonyls generated at 318°C, μg/mg-liquid				
PG	2.03 ± 0.80	2.35 ± 0.87	ND	1.3
GL	21.1 ± 3.80	2.4 ± 0.99	0.8 ± 0.5	12.9
PG/GL (1:1)	5.47 ± 0.72	1.76 ± 0.52	0.41 ± 0.05	4.6
E-liquid #1	5.99 ± 5.46	0.97 ± 0.87	0.05 ± 0.04	9.1
E-liquid #2	5.22 ± 4.35	1.37 ± 0.84	0.26 ± 0.13	5.6

*The results are expressed as mean ± one standard deviation*. Two to eight repeated experiments were conducted for each test.

ND: not detected. PG: propylene glycol. GL: glycerol.

### Implication for estimating carbonyl compound inhalation exposure from e-cigarettes

The amount of commercial e-liquid #2 consumed by ten 50-ml puffs using the purchased e-device with a refillable cartridge was measured to be around 43–48 mg. Based on the amounts of carbonyls generated from e-liquid #2 at different temperatures in the tubular reactor, the inhaled amounts of formaldehyde, acetaldehyde, and acrolein from ten 50-ml puffs at different temperatures were estimated (Table 3). From an online survey of e-cigarette users, Etter et al. [25] estimated the median number of puffs per day to be 175. Based on Table 3, the daily exposure to formaldehyde and acetaldehyde for an e-cigarette user vaping at 215°C could reach 105–117 μg and 36–42 μg, respectively. According to Geiss et al. [15], 215°C is within the most subjectively pleasant vapor generation temperature range. It is also within the peak working temperature range (138.6–231.0°C) of heating coil measured by Zhao et al. [22] This estimated daily formaldehyde exposure is well above the NSRL (No Significant Risk Level) of 40 μg/day from OEHHA (California Office of Environmental Health Hazard Assessment) [26] and the IRIS (Integrated Risk Information System) cancer risk value of 2.3 μg/day by U.S. EPA [27]. According to Counts et al. [28], the estimated formaldehyde in traditional tobacco smoke is up to 52 μg per cigarette or 8–10 puffs. If the e-cigarette heating temperature exceeds 270°C, the amount of formaldehyde generated from ten 50-ml puffs could reach similar levels (as shown in Table 3) as from traditional tobacco smoking. The cumulative effects of these toxic chemicals for long-term e-cigarette use warrant further study.

**Table 3:** Estimated carbonyls generated from 10 puffs (50 ml/puff, 3-s puff duration) of commercial e-liquid #2 at different temperatures.

Temperature	Formaldehyde, μg	Acetaldehyde, μg	Acrolein, μg
318°C	225–250	59–66	11–12
270°C	31–35	9–11	~1
215°C	6.0–6.7	2.1–2.4	ND
161°C	~1.4	ND	ND
108°C	~ 0.1	~1.2	ND
50°C	~ 0.4	ND	ND

ND: not detected.

## Conclusions

This study demonstrated that PG and GL are likely to be the main emission sources of carbonyls inhaled during e-cigarette use. Significant amounts of toxic carbonyl compounds can be created when common e-liquid solvents—i.e., PG, GL, or their mixture—are heated at high temperature, either intentionally by users to get more aerosol or accidently due to overheating. GL produced much more formaldehyde than PG under our testing conditions (over 27 times as much at 270°C, and over 10 times as much at 318°C), which could be due to the stainless steel reactor material in our study. Besides formaldehyde and acetaldehyde, detectable amounts of acrolein were also observed when reactor temperature exceeded 270°C and GL was present in the e-liquid. The authors realize that the results from a stainless steel tubular reactor may not exactly mimic what happens inside an e-cigarette. Nevertheless, this study developed a controllable test method to evaluate carbonyl emissions from different e-cigarette liquids under precisely controlled temperatures independent of the variation in specific e-cigarette devices in the market. The estimated daily exposure to formaldehyde at 215°C, exceeding USEPA and OEHHA acceptable cancer risk estimates for e-cigarette users, emphasizes the need to further examine the potential cancer and non-cancer health risks associated with e-cigarette use.
